# Clinical outcomes with oral cephalosporins as definitive treatment of Enterobacterales bacteremia from a urinary source based on cefazolin minimum inhibitory concentration

**DOI:** 10.1128/aac.01357-25

**Published:** 2026-02-18

**Authors:** Lauren Kobasuk, Tamara Cisowska, Karen Howard, Christian Gabriel, Chaorong Wu, Hannah Imlay, Emily S. Spivak, Kara Nazminia

**Affiliations:** 1 Department of Pharmacy, University of Utah Health 14434, Salt Lake City, Utah, USA; 2 Department of Internal Medicine, University of Utah School of Medicine 12348 , Salt Lake City, Utah, USA; University of California San Francisco, San Francisco, California, USA

**Keywords:** Enterobacterales bacteremia, cephalosporins

## Abstract

Cefazolin breakpoints differ for the treatment of Enterobacterales urinary tract infections and systemic infections. A retrospective, exploratory cohort study of 148 patients found no difference in 30-day treatment outcomes based on the blood culture cefazolin minimum inhibitory concentration (≤2 mcg/mL versus 4–16 mcg/mL) for patients transitioned to PO cephalosporins for the treatment of *Escherichia coli*, *Klebsiella pneumoniae*, or *Proteus mirabilis* bacteremia secondary to a urinary source. Further research should confirm these findings.

## INTRODUCTION

Transitioning patients with Enterobacterales bacteremia from intravenous (IV) to oral (PO) antibiotics is well supported by previous literature (1). The best approach for transitioning patients to PO therapy is not fully elucidated. Previous studies compared highly bioavailable agents like PO fluoroquinolones and trimethoprim-sulfamethoxazole to less bioavailable agents like PO beta-lactams and found no difference in clinical outcomes (2–4). However, one study found a higher bacteremia recurrence rate with PO beta-lactams (5). This prompted the evaluation of standard versus high dosing of beta-lactams for the treatment of Enterobacterales bacteremia (6, 7). Another factor that may contribute to varying treatment outcomes of patients with Enterobacterales bacteremia who were transitioned to PO cephalosporins is the minimum inhibitory concentration (MIC).

The Clinical and Laboratory Standards Institute (CLSI) recommends using cefazolin as a surrogate marker for PO cephalosporin susceptibility for the treatment of urinary tract infections (UTI) secondary to *Escherichia coli*, *Klebsiella pneumoniae*, and *Proteus mirabilis* (8). They further suggest differing susceptibility breakpoints for the treatment of uncomplicated UTI versus systemic infection, with a cefazolin MIC ≤16 mcg/mL and an MIC ≤2 mcg/mL considered susceptible respectively. This means a cefazolin MIC of 4-16 mcg/mL is considered sensitive for uncomplicated UTI but intermediate for bacteremia (8). Therefore, a urine culture may suggest susceptibility to PO cephalosporins based on the cefazolin MIC, while a blood culture susceptibility report may suggest the same organism is not susceptible to cefazolin based on an intermediate MIC. As clinicians may use either the urine or blood culture to guide transition to a PO agent for the treatment of bacteremia secondary to UTI, the evaluation of clinical outcomes when using an intermediately susceptible agent is warranted.

This retrospective, exploratory cohort study from January 2014 to January 2025 evaluated outcomes based on cefazolin MIC on the blood culture susceptibility report (≤2 mcg/mL for the “susceptible cohort” versus 4-16 mcg/mL for the “intermediate cohort”) for patients transitioned to a PO cephalosporin for the treatment of Enterobacterales bacteremia secondary to a urinary source. The University of Utah Institutional Review Board deemed this study exempt from review. Patients 18 years old with a matching urine and blood culture for *E. coli*, *K. pneumoniae*, or *P. mirabilis* collected within 24 h of each other who were transitioned to a PO cephalosporin within 5 days of index positive blood culture were included. Patients with a polymicrobial bacteremia, renal abscess, prostatitis, emphysematous pyelonephritis, mortality, or hospice transition during admission were excluded. Patients who received antibiotics with gram-negative activity for concomitant infection, greater than 14 days of antibiotics, or IV antibiotics after transition to a PO cephalosporin were also excluded. The oral cephalosporins in question may warrant renal dose adjustments for a creatinine clearance less than 30 mL/min. Patients with this degree of renal dysfunction were excluded, as these patients are less likely to be started on high-dose cephalosporin regimens.

The primary outcome was a composite of 30-day all-cause mortality, hospital readmission, emergency department (ED) visit, and recurrence of pyelonephritis or bacteremia. Recurrent bacteremia or pyelonephritis was defined as infection with the same initial organism in the blood or urine culture, respectively. Documentation of systemic symptoms in the chart was required for diagnosis of recurrent pyelonephritis. PO cephalosporin dosing regimens for the treatment of bacteremia were summarized. Dosing regimens were classified as either standard or high dose (Table 1).

**TABLE 1:** Cephalosporin dosing regimens

	Standard dose	High dose
Cephalexin	500 mg PO four times daily	1 g PO three times daily 1 g PO four times daily
Cefuroxime	250 mg PO two times daily	500 mg PO two times daily
Cefpodoxime	100 mg PO two times daily 200 mg PO two times daily	400 mg PO two times daily

Descriptive statistics were performed for baseline characteristics. For the difference between cohorts, Fisher’s exact test was applied to select categorical variables, while other categorical variables were assessed using the *χ*^2^ test. Continuous variables were analyzed using the Mann-Whitney U test. A *χ*² test was performed to evaluate the statistical significance of the observed difference in treatment outcomes between the two cohorts. A *p* value ≤ 0.05 was considered statistically significant. A power calculation was not performed, as this study included all eligible patients from a single center; therefore, the sample size was fixed by available data. We hypothesized that the susceptible cohort would have a lower risk of the composite primary outcome compared with the intermediate cohort.

After screening 228 patients, a total of 148 patients were included. There were 124 patients in the susceptible cohort (MIC ≤2 mcg/mL) and 24 patients in the intermediate cohort (MIC 4-16 mcg/mL). The majority of patients were excluded for a creatinine clearance of <30 mL/min (*n* = 18) or receiving greater than 14 days of antibiotic therapy (*n* = 17). Baseline characteristics are summarized in Table 2. The median duration of antibiotic treatment for bacteremia was 7 days in both cohorts, with most patients receiving 3 days of IV antibiotics before transitioning to a PO cephalosporin on day 4 (Table 3). Table 4 summarizes standard versus high dosing regimens in each cohort.

**TABLE 2:** Baseline characteristics

	Cefazolin MIC ≤2 mcg/mL (*n* = 124)	Cefazolin MIC 4-16 mcg/mL (*n* = 24)	*p* value
Female, *n* (%)	85 (68.5)	12 (50.0)	0.13
Age, median (IQR)	66.5 (53.0 to 76.0)	62.5 (54.2 to 71.0)	0.495
BMI (kg/m^2^), median (IQR)	28.2 (23.7 to 34.1)	28.5 (24.1 to 31.5)	0.675
CCI, median (IQR)	4.0 (2.0 to 8.0)	4.0 (1.8 to 6.0)	0.369
Cefazolin MIC on urine culture, *n* (%)	*n* = 123^*a*^	*n* = 24	<0.001
≤2 mcg/mL	112 (91.1)	1 (4.2)	
4-16 mcg	10 (8.1)	19 (79.2)	
≥32 mcg/mL	1 (0.8)	4 (16.7)	
Urologic complexities^*b*^^,*c*^, *n* (%)	23 (18.5)	6 (25.0)	0.574
Immunosuppression^*d*^, *n* (%)	12 (9.7)	0 (0)	n/a^*f*^
Steroids	2 (16.7)		
Transplant	6 (50.0)		
Active chemotherapy	4 (33.3)		
SIRS criteria, median (IQR)	3.0 (2.0 to 3.0)	3.0 (2.0 to 3.0)	0.598
ICU admission^*e*^, *n* (%)	31 (25.0)	5 (20.8)	0.861
Vasopressor use	14 (45.2)	2 (40.0)	1
ID consult^*a*^, *n* (%)	5 (4.0)	1 (4.2)	1

^*a*^ One patient in cohort 1 did not have a urine culture susceptibility report.

^*b*^ Defined as chronic indwelling catheter, urinary stone, renal calculus, urinary stent, or ureteral obstruction.

^*c*^ Fisher’s exact test used.

^*d*^ Defined as receiving steroid equivalence of prednisone ≥ 20 mg/day for ≥4 weeks, active chemotherapy, or recipient of solid organ transplant.

^*e*^ Admission to ICU at time of positive index blood culture.

^*f*^ n/a, not applicable.

**TABLE 3:** Antibiotic characteristics

	Cefazolin MIC ≤2 mcg/mL (*n* = 124)	Cefazolin MIC 4-16 mcg/mL (*n* = 24)	*p* value
IV antibiotic duration (days), median (IQR)	3.0 (2.0 to 3.0)	3.0 (3.0 to 4.0)	0.074
Switch to PO cephalosporin (day), median (IQR)	4.0 (3.0 to 4.0)	4.0 (3.0 to 5.0)	0.021
Total antibiotic duration (days), median (IQR)	7.0 (7.0 to 10.0)	7.5 (7.0 to 10.0)	0.809
Dosing regimen, *n* (%)			
High	93 (75.0)	12 (50.0)	0.026
Standard	31 (25.0)	12 (50.0)	

**TABLE 4:** Cephalosporin regimens

PO cephalosporin	Cefazolin MIC ≤2 mcg/mL (*n* = 124)	Cefazolin MIC 4–16 mcg/mL (*n* = 24)
Cefadroxil, *n* (%)	1 (0.8)	0 (0)
Standard^*a*^	1	
Cephalexin, *n* (%)	5 (4.0)	0 (0)
Standard	3	
High	2	
Cefuroxime, *n* (%)	92 (74.2)	8 (33.3)
Standard	5	6
High	87	2
Cefpodoxime, *n* (%)	24 (19.4)	16 (66.7)
Standard	20	10
High	4	6
Cefdinir, *n* (%)	2 (1.6)	0 (0)
Standard^*a*^	2	

^*a*^ All cefadroxil and cefdinir regimens were considered standard dose.

The composite primary outcome occurred in 26.6% of patients in the susceptible cohort compared to 20.8% of patients in the intermediate cohort (*X*^2^ = 0.35, 1, df = 1, *P* =0.05). Table 5 summarizes the primary outcome and its components.

**TABLE 5:** Counts and percentages of all outcomes stratified by blood culture MIC

	Cefazolin MIC ≤2 mcg/mL (*n* = 124)	Cefazolin MIC 4-16 mcg/mL (*n* = 24)	*p* value
Composite primary outcome, *n* (%)	33 (26.6)	5 (20.8)	0.55
30-Day mortality	0 (0)	0 (0)	
30-Day readmission	23 (18.5)	4 (16.7)	
30-Day ED visit	12 (9.7)	1 (4.2)	
30-Day bacteremia recurrence	3 (2.4)	0 (0)	
30-Day pyelonephritis recurrence	7 (5.6)	0 (0)	

While the composite outcome included all-cause events, very few ED visits or hospital readmissions were elucidated to be related to the prior infection on chart review. Out of the 12 ED encounters within 30 days of index blood culture in the susceptible cohort, only three were deemed related to the index hospitalization (recurrent pyelonephritis or cystitis). The only ED encounter reported in the intermediate cohort was unrelated to the index infection.

While the susceptible cohort was expected to have fewer adverse treatment outcomes, results did not support this hypothesis. Chart review suggests 30-day ED visits and readmissions—largely driving the higher composite outcome in the susceptible cohort—are often not related to the primary infection. Overall, this study adds to the literature supporting the transition to PO cephalosporins for the treatment of bacteremia secondary to UTIs from *E. coli*, *K. pneumoniae*, or *P. mirabilis*. In patients with contraindications to guideline-recommended PO agents, such as fluoroquinolones or trimethoprim-sulfamethoxazole, these results suggest PO cephalosporins might be an appropriate alternative, even when the cefazolin MIC is considered intermediate.

This study addressed an unexplored, yet possibly clinically significant question. To our knowledge, this is the first evaluation of treatment outcomes for patients transitioned to a PO cephalosporin for bacteremia based on cefazolin MIC. We also summarized cephalosporin dosing regimens, which is a unique strength of this study. Several limitations still exist. First, our evaluation was limited to a single-center retrospective evaluation including a small patient sample size, limiting generalizability and ability to detect true clinical differences between the groups. Also, adherence to prescribed cephalosporins in the outpatient setting could not be captured. Lastly, treatment outcomes could be underrepresented due to patients presenting to health systems that do not utilize a common electronic medical record system.

In summary, this study found no statistically significant difference in treatment outcomes based on the blood culture cefazolin MIC (≤2 mcg/mL versus 4-16 mcg/mL) in patients transitioned to a PO cephalosporin for the definitive treatment of *E. coli*, *K. pneumoniae*, or *P. mirabilis* bacteremia secondary to a urinary source. While this overall finding is promising, it remains exploratory, and further research involving a larger sample size is warranted.

## References

[1] Tamma PD, Conley AT, Cosgrove SE, Harris AD, Lautenbach E, Amoah J, Avdic E, Tolomeo P, Wise J, Subudhi S, Han JH, Antibacterial Resistance Leadership Group. 2019. Association of 30-day mortality with oral step-down vs continued intravenous therapy in patients hospitalized with enterobacteriaceae bacteremia. JAMA Intern Med 179:316–323. doi:10.1001/jamainternmed.2018.6226 30667477 PMC6439703

[2] Sutton JD, Stevens VW, Chang N-C, Khader K, Timbrook TT, Spivak ES. 2020. Oral β-lactam antibiotics vs fluoroquinolones or trimethoprim-sulfamethoxazole for definitive treatment of Enterobacterales bacteremia from a urine source. JAMA Netw Open 3:e2020166. doi:10.1001/jamanetworkopen.2020.20166 33030555 PMC7545306

[3] Saad S, Mina N, Lee C, Afra K. 2020. Oral beta-lactam step down in bacteremic E. coli urinary tract infections. BMC Infect Dis 20:785. doi:10.1186/s12879-020-05498-2 33087051 PMC7576740

[4] Mercuro NJ, Stogsdill P, Wungwattana M. 2018. Retrospective analysis comparing oral stepdown therapy for enterobacteriaceae bloodstream infections: fluoroquinolones versus β-lactams. Int J Antimicrob Agents 51:687–692. doi:10.1016/j.ijantimicag.2017.12.007 29284155

[5] Alzaidi S, Veillette JJ, May SS, Olson J, Jackson K, Waters CD, Butler AM, Hutton MA, Buckel WR, Webb BJ. 2024. Oral β-lactams, fluoroquinolones, or trimethoprim-sulfamethoxazole for definitive treatment of uncomplicated Escherichia coli or Klebsiella species bacteremia from a urinary tract source. Open Forum Infect Dis 11:ofad657. doi:10.1093/ofid/ofad657 38370295 PMC10873539

[6] Geyer AC, VanLangen KM, Jameson AP, Dumkow LE. 2023. Outcomes of high-dose oral beta-lactam definitive therapy compared to fluoroquinolone or trimethoprim-sulfamethoxazole oral therapy for bacteremia secondary to a urinary tract infection. Antimicrob Steward Healthc Epidemiol 3:e148. doi:10.1017/ash.2023.435 37771747 PMC10523554

[7] Punjabi C, Tien V, Meng L, Deresinski S, Holubar M. 2019. Oral fluoroquinolone or trimethoprim-sulfamethoxazole vs. ß-lactams as step-down therapy for enterobacteriaceae bacteremia: systematic review and meta-analysis. Open Forum Infect Dis 6:ofz364. doi:10.1093/ofid/ofz364 31412127 PMC6785705

[8] CLSI. 2014. Performance standards for antimicrobial susceptibility testing. Twenty-fourth informational supplement. Wayne, PA Clinical and Laboratory Standards Institute CLSI document M100-S24.

